# HIV-Related Immune Activation and Inflammation: Current Understanding and Strategies

**DOI:** 10.1155/2021/7316456

**Published:** 2021-09-29

**Authors:** Tingxia Lv, Wei Cao, Taisheng Li

**Affiliations:** ^1^Department of Infectious Diseases, Peking Union Medical College Hospital, Chinese Academy of Medical Sciences & Peking Union Medical College, Beijing 100730, China; ^2^Department of Infectious Diseases, Beijing Friendship Hospital, Capital Medical University, Beijing, China; ^3^Department of Basic Medical Sciences, School of Medicine, Tsinghua University, Beijing 100084, China; ^4^Tsinghua-Peking Center for Life Sciences, Beijing 100084, China

## Abstract

Although antiretroviral therapy effectively controls human immunodeficiency virus (HIV) replication, a residual chronic immune activation/inflammation persists throughout the disease. This aberrant immune activation and inflammation are considered an accelerator of non-AIDS-related events and one of the driving forces of CD4^+^ T cell depletion. Unfortunately, HIV-associated immune activation is driven by various factors, while the mechanism of excessive inflammation has not been formally clarified. To date, several clinical interventions or treatment candidates undergoing clinical trials have been proposed to combat this systemic immune activation/inflammation. However, these strategies revealed limited results, or their nonspecific anti-inflammatory properties are similar to previous interventions. Here, we reviewed recent learnings of immune activation and persisting inflammation associated with HIV infection, as well as the current directions to overcome it. Of note, a more profound understanding of the specific mechanisms for aberrant inflammation is still imperative for identifying an effective clinical intervention strategy.

## 1. Introduction

With the development of well-tolerated and highly effective antiretroviral therapy (ART), HIV/AIDS has changed from a fatal disease into a chronic and controllable condition [1]. As a result, the life expectancy of HIV-infected patients with a high CD4^+^ T cell count and an undetectable viral after ART is gradually approaching that of the uninfected population [2]. However, abnormal immune activation and inflammation are accompanied by the whole infection process, and antiviral therapy alone is challenging to solve these clinical problems. For example, people living with HIV (PLWH) after successful ART still showed a higher level of immune activation, characterized by elevated biomarkers such as IL-6, D-dimer, C-reactive protein (CRP), and sCD14 [3, 4]. Chronic immune system activation is a hallmark of HIV infection and better predicts disease outcome than plasma viral load [5]. However, clinical data suggested that only up to 30% of patients after ART present a modest rise of CD4^+^ T cell levels, far from effective immune reconstitution. Moreover, levels of inflammation are associated with disease progression in PLWH [6–8], which predicts an accelerated and accentuated onset of serious non-AIDS events (SNAEs), such as neurocognitive disorders, coronary artery disease, chronic liver/kidney dysfunction, metabolic syndrome, osteoporosis, and non-HIV-associated cancers [8, 9]. As a result, patients remain susceptible to opportunistic infections and are more prone to disease progression and poor outcomes in this setting.

Admittedly, the persistence of the HIV-1 reservoir after ART is an important reason for HIV-related immune activation and inflammation [10, 11]. However, HIV-related immune activation and inflammation are a systematic and long-term process, and many other factors and mechanisms are also involved. Given its complexity and burden for patients, treatment for abnormal immune activation and inflammation has gradually become indispensable, and clinical trials using anti-inflammatory properties are underway to eliminate this phenomenon. This review will systematically elucidate the current mechanisms and therapeutic strategies/drugs for HIV-related immune activation and inflammation.

## 2. Driving Factors for HIV-Related Immune Activation

After the antigenic stimulation of the HIV, T cell is activated and induces the innate and adaptive immune response. However, the immune activation continues even after the HIV-1 viral load decreased to an undetectable level. The underlying mechanisms of persistent immune activation are complex, and various factors have been proposed to date.

### 2.1. The Persistence of HIV Viral Reservoirs

Continuous production of viral particles by reservoir cells in ART patients remains an important source of immune activation [12]. Admittedly, viral load and inflammation were decreased and CD4^+^ T cell elevated after ART [13]. However, after combined antiretroviral therapy (cART), a small amount of HIV in the reservoirs cannot be eliminated. Consequently, its expression products activate lymphocytes and macrophages to cause immune activation. For example, HIV envelope glycoprotein 120 (gp120) induces IL-1*β* release from macrophages through binding to the chemokine receptor CCR5 and coupling to G(i)alpha protein [14]. HIV-1 structural proteins, p17, p24, and gp41, act as viral pathogen-associated molecular pattern (PAMP) signaling through TLR2 and its heterodimers leading to significantly increased immune activation via the NF*κ*B signaling pathway [15]. Likewise, HIV-1 ssRNA interacts with the pattern recognition receptors TLR-7 and TLR-9 in plasmacytoid dendritic cells and induces the production of IFN-*α* and may therefore contribute to chronic immune activation [16]. Thus, HIV-1 persistence in individuals is at an increased risk for developing non-AIDS-related comorbidities [17, 18]. It is reported that pretreatment CD8^+^ T cell activation, nadir CD4^+^ T count, and CD4 : CD8 ratio might predict reservoir size [19]. Studies also have shown that administration of integrase inhibitors could partially reduce the reservoirs, decrease the number of infected memory CD4^+^ T cells, and significantly decrease immune activation levels such as CD38^+^CD8^+^ T cells [20–22]. Based on the current understanding of HIV, therapy targeting HIV repositories is undoubtedly one of the critical strategies.

### 2.2. Intestinal Microbial Translocation

The disturbance in gut microbiota has been widely reported in HIV-infected subjects compared with healthy controls [23, 24]. Generally, HIV destroys the CCR5^+^CD4^+^ T cells, which are abundantly present in the gut-associated lymphoid tissues, leading to the destruction of the intestinal mucosal barrier. Then, many intestinal microbial and their metabolites entered the blood circulation, inducing immune activation and hyperinflammatory response [25, 26]. Circulating lipopolysaccharide (LPS), an indicator of microbial translocation, was significantly increased in chronically HIV-infected individuals and simian immunodeficiency virus- (SIV-) infected rhesus macaques [5, 27]. The microbial translocation is also associated with the proportion of CD8^+^ T cells overexpressing CD38, IFN concentration in the blood [26], and sustained failure during CD4^+^ T cell reconstitution in treated patients [28]. On the other hand, the high inflammatory state of intestinal mucosa and apoptosis of intestinal epithelial cells further promoted intestinal microbial translocation [29]. Therefore, dysregulation of intestinal microbial and microbial translocation is significantly correlated with immune and inflammatory activation [30].

### 2.3. Depletion of Regulatory T (Treg) Cells

The distribution of Treg cells, the potent natural regulator, changed during chronic HIV infection and was accompanied by a shift of CD4^+^CD25^+^ Treg from the peripheral blood to peripheral lymph nodes and mucosal lymphoid tissues [31–33]. Treg cells can inhibit T cell activation and proliferation through contact-dependent mechanisms, but Treg cell itself is vulnerable to HIV infection, leading to a significant functional deficit [34, 35]. Treg cells were also reported to control immune activation during HIV-1 infection through impairing IL-2 production [36]. Even in HIV elite controllers, the decreased Treg cells are strongly associated with immune activation and can increase the incidence of atherosclerosis and other related inflammatory diseases [37, 38]. In other words, growing Treg cells may reduce HIV-associated immune activation, which is the basis of current clinical trials using statins to suppress immune activation [39].

### 2.4. Coinfection with Other Viruses

Compared to HIV-monoinfected individuals, patients with other virus infections have elevated soluble inflammatory biomarkers and surface markers of T cell activation [40–43]. HIV infection also leads to the lower immune function of the body and often causes the reactivation of some viruses that have been lurking in the body for a long time, such as cytomegalovirus (CMV) and human herpesvirus (HSV). Margolick et al. showed that in well-controlled HIV-infected patients, the body's specific T cell response to CMV would affect chronic immune activation [44]. Active HSV infection, regardless of symptoms, involves the recruitment of activated CD4^+^ T cells to the genital area and can lead to breaks in the mucosal layer through which HIV can enter [45, 46]. HIV-1 infection also impairs HSV-specific CD4^+^ and CD8^+^ T cell response by reducing Th1 cytokines and CCR5 ligand secretion [47], while HSV-2 infection significantly increases the susceptibility of the host to acquire HIV and promotes the shedding of the latter during the coinfection [48]. In addition, in patients coinfected with HIV and HCV, the inflammatory response, platelet activation, and oxidative stress are more intense, indicating its enhanced immune activation [49, 50].

## 3. Pathogenesis of HIV-Related Inflammation

### 3.1. Toll-Like Receptors (TLRs) and Nuclear Factor-Kappa B (NF-*κ*B) Activation

Toll-like receptors (TLR1-10) are pattern recognition receptors (PRRs) expressed on the surface or inside various immune cells, which could recognize multiple PAMPs such as LPS, peptidoglycan, virus, and nucleic acid [51]. HIV single-stranded RNA (ssRNA) and double-stranded RNA (dsRNA) are formed during their life cycle and can be recognized by intracellular TLRs. For example, TLR7 recognizes the HIV ssRNA, leading to the destruction of T cells and the release of inflammatory cytokines [52]. Inflammatory activation caused by TLR3 recognition of HIV dsRNA is linked with HIV-associated blood-brain barrier disorders and neurological dysfunction [53]. HIV infection can also lead to the expression changes of TLRs. For example, TLR3 and TLR4 are not expressed in T cells of healthy people, but their expression is significantly increased after HIV infection [54]. HIV-1 ssRNA stimulation of neutrophils leads to enhanced expression of TLR7/8, RIG-I, and MDA5, decreased expression of TLR2, induction of cytokines (TNF-*α* and IL-6), and production of ROS [55]. In addition, after HIV infection, the gp120 activates IKK*β*, which subsequently leads to endogenous I*κ*B*α* phosphorylation, nuclear translocation of NF-*κ*B, and then overexpression of IL-6 and IL-8 [56]. HIV-associated neurocognitive disorders (HAND), which exist in approximately 50% of infected individuals even after a highly active antiretroviral therapy, correlate with the activation of NF-*κ*B [57]. For example, in central nervous system lesions, HIV transactivator of transcription (Tat) protein induces the expression of proinflammatory genes in astrocytes, which ultimately activates NF-*κ*B and upregulates the expressions of MCP-1, IL-8, CXCL10, etc. [58]. Additionally, decrement in excitatory amino acid transporter 2 (EAAT-2) in astrocyte plasma membranes leads to elevated levels of extracellular glutamate, while this increased EAAT-2 inhibition via the NF-*κ*B signaling pathway during HAND [59].

### 3.2. Interferon and Interferon-Stimulated Genes

HIV triggers the induction of type I IFN (IFN-*α*/*β*), providing a crucial mechanism of antiviral defense and inflammation response [60, 61]. Interferon regulatory factors also participate in the induction of type I interferon, and interferon-stimulated genes (ISG) produce many subsequent effects [62]. It was reported that IFN-*α*/*β* binding to cell surface receptors induces the expression of ISG-15 by activating the downstream JAK-STAT pathway [53, 63]. The expression of ISG-15 was positively correlated with plasma viral load and CD4^+^ T cell count in HIV-1 chronically infected patients [64]. Moreover, ISG-15 increases the expression of IP-10, a critical inflammatory factor in HIV-induced immune dysfunction and disease progression, by activating NF-*κ*B, suggesting the crosstalk between the NF-*κ*B and interferon signaling [65]. In addition, the expression of IP-10 is also negatively regulated by miR-21, and the increased expression of ISG-15 can also reduce the inhibitory effect of miR-21 on IP-10 [66].

### 3.3. Cysteinyl Aspartate-Specific Protease- (Caspase-) Induced Cell Apoptosis and Pyroptosis

Caspase family participates in apoptosis/pyroptosis under various stimulation, such as cytokines and DNA damage, of which caspase-3/8/9 is the main effector of cell apoptosis and caspase-1 mediated the pyroptosis [67]. HIV-infected cells exhibited increased programmed cell death, such as apoptosis, pyroptosis, and ferroptosis than uninfected cells [68]. The decrease of CD4^+^ T cells can be simultaneously mediated by apoptosis and pyroptosis, while the immune activation is more closely related to pyroptosis during HIV infection [69, 70]. It is reported that the main pattern of CD4^+^ T cell death caused by HIV is caspase-1-mediated pyroptosis (about 95%), while the proportion of cell apoptosis is less than 5% [70]. In HIV-infected patients without ART, caspase-1/3 expression in CD4^+^ T cells and caspase-3 expression in CD8^+^ T cells were significantly increased [71]. Meanwhile, there was a positive correlation between HLA-DR^+^CD38^+^CD8^+^ T cells and CD4^+^ T cells with high expression of caspase-1 [71]. In addition, caspase-1-mediated cell apoptosis plays a vital role in the occurrence and development of immune reconstitution inflammatory syndrome (IRIS), as evidenced by the increased serum cytokine levels and caspase-1/5 in HIV-associated tuberculosis infection patients with IRIS [72]. Besides cell death, caspases are also centrally involved in inflammation responses, among which the secretion of IL-1*β* and IL-18 plays a key role [73]. After HIV infection, bystander CD4^+^ T cells produce HIV-1 DNA, which is recognized by the host DNA receptor interferon-*γ*-inducible protein 16 (IFI16) and then binds to apoptosis-associated speckle-like protein (ASC) and procaspase-1 to form a functional inflammasome [74]. Then, the inflammasome mediated the cleavage and mature of pre-IL-1*β* and pre-IL-18. Meanwhile, the activated caspase-1 also leads to increased cell permeability, edema/rupture, and the release of other damage-related molecular patterns (DAMP) such as HMG-B1, IL-33, and inflammatory cytokines [67, 70, 71]. It is worth mentioning that the transmission of HIV between cells promotes pyroptosis, while free virus particles could not induce pyroptosis of CD4^+^ T cells [75].

## 4. Strategies to Reduce HIV-1 Related Immune Activation and Inflammation

Currently, the strategies aimed at coping with HIV-1-related immune activation and inflammation are still challenging due to the complexity of immune activation, the differences of the PLWH population, and uneven inclusion criteria. However, some drugs are emerging in the experimental stage though there is still a long way to go before eradicating immune activation.

### 4.1. Immunosuppressive Drugs

#### 4.1.1. Prednisolone

In 2012, Kasang et al. reported that prednisolone has beneficial effects on immunological correlates of HIV disease progression in untreated HIV-infected patients (CD38^+^CD8^+^%, CD38^+^CD4^+^%, sCD14, and LPS-binding protein (LBP)), but no additional beneficial effects in patients treated with ART [76]. Consistent with this viewpoint, Wallis et al. reported that no effect on markers of cell activation or apoptosis after eight weeks of prednisone (40 mg/d) treatment as an adjunct to ART in 24 HIV-infected subjects with >200 CD4^+^ T cells/*μ*L [77]. Similarly, a randomized clinical trial of prednisolone (5 mg/day, two years) applied in ART naïve patients showed a significant decrease in immune activation (sCD14, suPAR, and CD38/HLA-DR/CD8^+^) and an increase in CD4^+^ T counts, while no significant effect on the primary endpoint of HIV disease progression to AIDS although viral load increased quickly than placebo [78]. Therefore, these facts might illuminate that prednisolone is more possibly associated with a stabilization of CD4^+^ T cell count [79, 80], which predicted the promising application of prednisolone in abnormal immune activation after clearance of HIV.

#### 4.1.2. Chloroquine/Hydroxychloroquine (CQ/HCQ)

Agents with immunosuppressive properties were first tried in PLWH in 1998, which could both break the vicious cycle that leads to the slow attrition of the lymphocyte pools [81]. Both CQ and HCQ have been widely used in treating autoimmune conditions, such as lupus and arthritis, and they could also suppress the HIV-1 replication in patients [82]. In 2010, Murray et al. reported the combination usage of CQ with ART in PLWH and indicated a reduced activation and proliferation of memory CD8^+^ T cells (proportions of HLA-DR, CD38, and Ki67 expression), as well as plasma LPS levels [83]. Piconi and colleagues also reported that HCQ (400 mg daily) reduced the level of activated CD4^+^(CD4^+^/Ki67^+^), CD14^+^(CD14^+^/CD69^+^) T cells, and IL-6/TNF-*α*, but increased the proportion of Tregs [84]. However, a placebo-controlled trial in the United Kingdom in 2012 showed that among HIV-infected patients not taking ART, 48 weeks of HCQ monotherapy (400 mg/d) did not reduce CD8^+^ T cell activation and IL-6/D-dimer level but did result in a more significant decline in CD4^+^ T cell count and increased viral replication [85]. Furthermore, Routy et al. conducted an open-labeled single-arm study and found no substantial changes in the levels of immune activation or inflammation markers (HLA-DR, CD38, and IL-6) after 24 weeks of ART+CQ (250 mg/d) [86]. Therefore, the role of CQ/HCQ in HIV-induced immune activation/inflammation is limited, especially in the presence of HIV. Of note, the conflicting results among different studies might be partially derived from the insufficient dosage of CQ/HCQ.

#### 4.1.3. Cyclosporin A (CsA)

Clinical trials suggested that CsA treatment was associated with modest but transient increases in the CD4^+^ T count and delayed progression to AIDS [87–89]. Cyclosporin treatment also significantly decreased the production of various cytokines (IL-2, TNF-*α*, and IFN-*γ*) in vitro [90]. Rizzardi et al. enrolled nine patients with primary HIV infection to receive CsA (0.3-0.6 mg/kg) in conjunction with ART for eight weeks, after which CsA was discontinued and ART continued for another 58 weeks. Results showed that patients with CsA treatment experienced a significant rise in CD4^+^ T count compared to the HAART-only control. Additionally, the proportion of IFN-*γ*-secreting CD4+CCR7-T cells was significantly higher in the CsA group during treatment [91]. However, it is essential to underscore that CsA must be used with ART; otherwise, patients' conditions might deteriorate quickly [89, 92, 93].

#### 4.1.4. Rapamycin (RAPA)

As known to date, RAPA is a macrocyclic lactone antibiotic with immunosuppressive properties and is currently used for prophylaxis of rejection in patients following organ transplantation. RAPA exerts its immunosuppressive function by inhibiting the mammalian target of rapamycin (mTOR) and further prevents cytokine-mediated T cell proliferation [94, 95]. In addition, RAPA can repress HIV-1 replication and reduce the release of IL-8 and MIP-1*α* in PBMC induced by *α*CD3/*α*CD28 [90, 96]. Coadministration of RAPA with CsA achieved a more obvious suppressive effect for HIV-1 reactivation in vitro and inhibited the production of cytokines such as IL-2, MCP-1, MIP-1*α*, IL-1*β*, IFN-*γ*, TNF-*α*, and IL-6 [90]. The first clinical trial of RAPA was conducted in six HIV-infected individuals (CD4^+^ T cell count > 100/*μ*L and VL < 50 copies/mL) who received liver transplantation, and results showed that patients who switched to RAPA monotherapy present improved control of HIV but no benefit in maintaining a higher CD4^+^ T cell count compared to those treated with calcineurin inhibitors [97].

#### 4.1.5. Tripterygium wilfordii Hook F (TwHF)

As a traditional Chinese medication, TwHF has been widely used to treat different autoimmune diseases, including rheumatoid arthritis and active Crohn's disease [98, 99]. Among the significant bioactive component extracted from TwHF, triptolide has been demonstrated to reduce LPS-induced inflammation [100–103]. Li et al. creatively combined TwHF (10 mg, three times/d) with ART in 18 immune nonresponder HIV patients. After one year of treatment by TwHF extract, CD4^+^ T cell count markedly increased, and the percentage of CD38^+^HLA-DR^+^ expressed on CD8^+^ T and CD4^+^ T cells decreased significantly during the 12-month treatment period [104]. Given these promising results, randomized placebo-controlled studies that enrolled more patients are warranted to evaluate the effects of TwHF extract on HIV patients.

### 4.2. Stains

Statins, including atorvastatin, fluvastatin, lovastatin, pitavastatin, pravastatin, rosuvastatin, and simvastatin, were generally used to decrease cholesterol. In recent years, statins have been tried for controlling HIV-associated inflammation but yielded mixed results. Ganesan et al. conducted a study in which 24 HIV-1-infected and ART naïve adults were enrolled to receive either atorvastatin 80 mg daily or placebo for eight weeks (phase A), followed by a washout phase of 4-6 weeks and a subsequent switch to complete additional 8 weeks (phase B) in the opposite assignment. The results showed that atorvastatin reduced the proportions of circulating CD4^+^HLA-DR^+^ (-2.5%), CD8^+^HLA-DR^+^ (-5%), CD8^+^HLA-DR^+^CD38^+^ T cells (-3%) [105]. In contrast, in another study by Eckard et al., no statistically significant difference was observed in the percentage of hsCRP, IL-6, sTNFR-I/II, IP10, and D-dimer between rosuvastatin (10 mg daily for 24 weeks) and placebo at initial analysis [106]. However, the secondary analysis demonstrated that rosuvastatin reduced sCD14, Lp-PLA2, and IP-10 levels over 48 weeks, with a greater decrease in the proportion of activated (CD38^+^HLA-DR^+^) T cells between the arms (−38.1% *vs.* −17.8%, or CD4+ cells and −44.8% *vs.* −27.4% for CD8+ cells) [107]. It is worth noting that the safety and drug-drug interactions regarding stains should be carefully considered. For example, atorvastatin appears to be relatively safe for long-term use at submaximal doses if monitored, while pravastatin, rosuvastatin, and pitavastatin appear to have the most benign safety profiles among statins when coadministered with ART and may not require dose adjustment [108].

### 4.3. Treatment on Microbial Translocation

#### 4.3.1. Probiotics/Prebiotics

Given the crucial role of decreasing bacterial translocation and proinflammatory cytokine production in the maintenance of gut homeostasis, new therapies aimed at restoring the integrity of the epithelial and gut-associated lymphoid tissue (GALT) through oral prebiotics, probiotics, or synbiotics are promising for alleviating HIV-related disease progression. Disturbance in gut microbiota has been widely reported in HIV-infected subjects, and specific intestinal microbiota might benefit HIV-infected patients during ART by improving the microbiota composition and reducing mucosal and systemic inflammation [109, 110]. Probiotics/prebiotics was partially reported to improve gastrointestinal immunity in SIV-infected macaques [111] and decrease microbial translocation and immune activation in ART-treated HIV-infected individuals [109, 112, 113]. However, the results were not always encouraging. Hummelen et al.'s study enrolled 32 women infected with HIV and given probiotic supplementation for 25 weeks; the results showed that changes in IFN-*γ*, IL-10, IgG, and IgE did not differ from the placebo group, which is consistent with Villar-García et al.'s study [110, 114]. Additionally, based on the potential benefits of intestinal microorganisms, food additives such as vitamin [115], recombinant lactoferrin [116], and Mediterranean diet are also being explored in HIV patients [117]. In summary, the evidence for the efficacy of probiotics, prebiotics, and synbiotics in control inflammation as presented in current studies is insufficient, and further comprehensive studies are needed to reveal their exact effect.

### 4.4. Hypoglycemic Agents

Some hypoglycemic agents have been found to benefit microbiota composition, promote gut barrier integrity, and reduce inflammation in human and animal models of diabetes. For example, treatment with metformin in PLWH alleviated lipodystrophy syndrome, hyperlipidemia, and insulin sensitivity. Moreover, metformin prevented the progression of coronary artery calcification and calcified plaque volume in PLWH with metabolic syndrome [118]. In 6 nondiabetic PLWH, metformin decreased CD4^+^ T cell expression of the marker of cell exhaustion programmed cell death-1 (PD-1) but not T cell activation markers CD38 and HLA-DR [119]. CD4^+^ T cell counts, CD4^+^/CD8^+^ T cell ratios, and plasma markers of inflammation/gut damage underwent minor variations in the blood in response to metformin [120]. Furthermore, treatment of nondiabetic individuals with metformin controls inflammation by improving glucose metabolism and by regulating intracellular immunometabolic checkpoints [121]. Another hypoglycemic agent, sitagliptin, was previously reported to reduce inflammation and chronic immune cell activation in cART-treated HIV-infected adults with impaired glucose tolerance. After eight weeks of sitagliptin administration, plasma hsCRP and CXCL10 concentrations and adipose tissue MCP-1 abundance were significantly declined than placebo, while the CD4^+^/CD8^+^ helper/suppressor ratio, D-dimer, and IL-6 concentrations were not significantly different [122]. Based on these preliminary but various results, large-scale and long-term studies are needed to determine whether hypoglycemic agents reduce cardiovascular risk and events in HIV-infected adults.

### 4.5. Other Drugs

#### 4.5.1. Antiplatelet Agents

Increased platelet activity has been reported in ART-treated HIV infection, and in vitro studies showed that HIV-1 plasma could activate healthy platelets, which in turn activated monocytes, implicating a direct role for activated platelets in immune activation [123]. In addition, a higher thrombogenicity and inflammation/immune activation contribute to the increased cardiovascular disease risk in PLWH [124]. Therefore, antiplatelet agents were tried in ART-experienced patients. For example, one week of low-dose aspirin treatment for ART-treated HIV-1-infected subjects exhibited a decreased activation marker of T cell (CD38 and HLA-DR) and monocyte (sCD14), as well as enhanced leukocyte responses to Toll-like receptor stimulation [123]. Unfortunately, the latest result published in 2019 showed that aspirin administration 81 mg daily in addition to ART did not benefit from decreasing inflammation, while another platelet inhibitor, clopidogrel, exhibited anti-inflammatory activity in PLWH [124]. Despite the mixed results to date, traditional interventions using antiplatelet agents to reduce CVD risk in HIV have been one choice, considering the overwhelming evidence that increased platelet activation is associated with an increased risk of cardiovascular events in PLWH.

#### 4.5.2. Cyclooxygenase Type 2 (COX-2) Inhibitor

Cyclooxygenase is a critical-step enzyme in the inflammatory process, which results in the direct production of inflammatory mediators. Celecoxib, a COX-2 inhibitor, has been used for 12 weeks in PLWH without ART by Kvale's group in Norway and showed reduced CD38^+^CD8^+^ T%, PD-1^+^CD8^+^ T%, IgA levels, and enhanced Treg number [125]. In 2017, this group published another study based on a novel COX-2 inhibitor etoricoxib, which also obtains a sound effect for reducing activation of CD8 T cells and improving Gag-specific T cell responses in ART naïve patients. However, in this study, etoricoxib does not modulate soluble markers of inflammation (sCD25, IP10, CD163, CD14, IL-6, and CRP), and in a surprise twist, no significant immunological effects were observed in ART-treated patients [126].

#### 4.5.3. Medical Cannabis

Previous human studies suggested that cannabis, to some extent, reduced the related symptoms (anorexia, cachexia, and neuropathic pain) and morbidity/mortality in PLWH [127, 128]. As for immune activation and inflammation in HIV patients, Manuzak et al. clarified that heavy cannabis users had decreased frequencies of HLA-DR^+^CD38^+^CD4^+^ and CD8^+^ T cell, increased frequencies of classical monocyte subsets (CD14^++^CD16^−^), and reduced frequencies of IL-23 and TNF-*α* producing antigen-presenting cells [129]. Moreover, recent cannabis use was associated with lower levels of inflammatory biomarkers, in both cerebrospinal fluid (CSF) and blood, suggesting its specific antineuroinflammatory effects [130]. Although there are promising benefits of cannabis for HIV/AIDS sufferers, the potential psychoactive side effects (impaired memory, euphoria, anxiety, and paranoia) and minor nonpsychoactive effects (sleepiness, tiredness, dry mouth, and red eyes) continue to be a barrier to its medical use. Also, the similar drug heroin was being tried in PLWH for treating immune activation and cardiovascular risk in HIV (NCT03976258), but no study results were posted to date.

Besides, following treatment of pyridostigmine, an ACh-esterase inhibitor, in 9 treatment-naïve HIV-1 patients with CD4^+^ T cell count over 300 cells/*μ*L, the fraction of CD69^+^CD4^+^ T cells, IFN-*γ*, and TNF-*α* was significantly decreased, and Treg was dampened, while IL-4/6/10 were increased compared with placebo [131]. Dipyridamole was demonstrated to inhibit the replication of HIV-1 [132]; however, in virally suppressed persons with HIV on ART, it did not decrease the soluble markers of inflammation levels but modestly reduced the levels of CD8^+^ T cell activation [133]. Administration of mesalazine to subjects with poor CD4^+^ T cell gain on virologically suppressive cART did not affect markers of peripheral inflammation [134]. Moreover, leflunomide was proven to reduce the proliferation of activated T cells in vitro [135], but when applied in a small RCT in cART-naive patients, it showed no significant changes in activated CD4^+^ and CD8^+^ T cells [136]. Finally, the potential benefits for immune activation/inflammation among HIV-1-infected subjects of other drugs, such as isotretinoin (NCT01969058) and methotrexate (NCT01949116), require additional investigation [137].

## 5. Conclusion and Perspective

In conclusion, though many pathogeneses of HIV-related immune activation and inflammation, such as HIV-1 reservoir, coinfections, and various inflammatory signaling, have been clarified, the current understanding for this complex disease does not meet the need to develop specific therapeutic approaches. Moreover, non-AIDS-related events also accelerated the disease progression. Currently, though numerous approaches and strategies have been proposed for curing HIV (Table 1), no scalable solution has yet been reached. Therefore, on the one hand, a further understanding of the specific pathogenesis or their interaction causing aberrant HIV-associated immune activation/inflammation and effective intervention strategies is still imperative. On the other hand, drug intervention at the very early stage before the HIV reservoir is composed might be a promising strategy.

## Figures and Tables

**Table 1:** Current Interventions for HIV-related Immune Activation and Inflammation.

Drug	Main inclusion criteria/groups	Case/control	Dose	Country	Efficiency/mechanism	Reference
Immunosuppressive drug						
Prednisolone	CD4 > 300 cells/*μ*L, absence of AIDS-defining symptoms, ART-naïve patients	Prednisolone (*n* = 163)/placebo (*n* = 163)	5 mg, po, qd, 2 years	Tanzania & Germany	sCD14 ↓, CD38/HLA-DR/CD8^+^ ↓, CD4 count ↑, HIV viral load ↑	[78]
Prednisolone	5 groups: (1) HIV-1 subjects untreated; (2) HIV-1 subjects treated with prednisolone; (3) HIV-1 with ART; (4) HIV-1 with ART+ prednisolone; (5) elite controllers	Untreated (*n* = 10); prednisolone (*n* = 27); ART (*n* = 30); prednisolone+ART (*n* = 31); elite controllers (*n* = 3)	5 mg/day	Germany	CD38^+^CD8^+^% ↓, CD38^+^CD4^+^% (-), sCD14 ↓, LPS-binding protein (LBP) ↓, ART group vs. ART+prednisolone group: CD38^+^CD8^+^% (-), D38^+^CD4^+^% (-), sCD14 (-), LBP (-)	[76]
HCQ	ART-treated HIV patients, CD4 < 200 cells/L during the last 12 months of therapy, VL < 37 HIV RNA copies/mL	Prior/posttreatment (*n* = 20)	400 mg/day, 6 w	Italy	Ki67CD4% ↓, CD69CD14% ↓; IL-6/TNF*α* ↓, Tregs↑, IFN-*α* secreting plasmacytoid DC ↓; LPS/TLR-mediated immune activation ↓; HLADRII, CD69, and CD38/CD45RO CD8 T% ↓	[84]
HCQ	18 to 65 years, naive to ART or no therapy in the previous 12 months; CD4^+^T ≥ 400 cells/*μ*L, VL ≥ 1000 copies/mL	HCQ (*n* = 42)/placebo (*n* = 41)	400 mg, 48 w	UK	CD8^+^ T cell activation (-), CD4^+^ T cell activation (-), D-dimer (-), IL-6 (-), Ki67^+^CD4, Ki67^+^CD8, CD4 cell count ↓	[85]
CQ	Off-ART (arms A and B): HIV-1-infected; on-ART (arms C and D) participants: ART ≥ 24 months, VL < 400 copies/mL, CD4 cell count < 350 cells/*μ*L	Arm A (*n* = 16); arm B (*n* = 17) Arm C (*n* = 18); arm D (*n* = 19)	Arms A and C: CQ (250 mg,12 w)/placebo (12 w); arms B and D: placebo (12 w)→CQ (250 mg, 12 w)	US	On-ART cohort: HLA-DR^+^CD8^+^ ↓, CD38^+^CD8^+^ ↓, IP10 ↓	NCT00819390
Cyclosporine	Primary HIV infection (HIV-1 antibody negative, HIV-1 RNA positive in plasma, and ≥3 bands in western blot)	CsA+ART (*n* = 9), ART (*n* = 29)	0.3-0.6 mg/kg po, q12 h, 8 w	Switzerland, Italy	CsA+HAART constantly maintained higher levels of CD4+ T cells; week 48: HIV-1-specific IFN-*γ*-secreting CD4 T% is higher than ART alone cohort	[91]
TwHF	cART ≥ 2 years, plasma HIV-1 VL < 40 copies/mL and suboptimal CD4 cell recovery	INRs (*n* = 9)/inadequate responders (*n* = 9)	10 mg, po, tid, 12 months	China	Both groups: CD4 T cell count ↑, CD38^+^HLA-DR^+^CD8 T cell% ↓, CD38^+^HLA-DR^+^CD4 T cell% ↓	[104]
Stains						
Atorvastatin	No ART, CD4^+^T > 350/*μ*L, HIV-1 RNA > 1000 copies/mL, LDL < 130 mg/dL	Atorvastatin (*n* = 34)/control (*n* = 24)	80 mg, qd, 8 w	US	CD4^+^HLA-DR^+^% ↓; CD8^+^HLA-DR^+^% ↓; CD8^+^HLA-DR^+^CD38^+^ T cells% ↓, CD4T (-), CD4^+^HLA-DR^+^CD38^+^ T cells% (-), CD4^+^CD38^+^% (-), CD8^+^CD38^+^% (-) TC ↓, LDL ↓	[105]
Rosuvastatin	ART duration ≥ 6 months, HIV-1 RNA < 1000 copies/mL, LDL cholesterol ≤ 130 mg/dL; hsCRP ≥ 2 mg/L and/or expression of CD38 and HLA-DR antigens ≥19% of CD8^+^ T cells at screening	Statin (*n* = 72)/placebo (*n* = 75)	10 mg, po, qd	US	hsCRP, IL-6, sTNFR-I/II, IP10 and D-dimer (-) Lp-PLA2 level ↓; DL cholesterol level ↓	
Treatment on microbial translocation						
Probiotic	Women, 18–45 years, not-normal vaginal microbiota, no ART (CD4 count > 200 cells/*μ*L)	Probiotics (*n* = 32)/placebo (*n* = 33)	Lactobacillus rhamnosus GR-1 and Lactobacillus reuteri RC-14 (2 × 10^9^ colony forming units), bid, 25 w	London, Netherlands	Insignificant changes in the immune parameters IFN-*γ*, IL-10, IgG, and IgE	[110]
Synbiotic dietary supplement	Adult females, currently taking ART, CD4 count > 200 cells/*μ*L	Synbiotic (*n* = 14)/fiber group (*n* = 13)	SynBiotic 2000®, fibers, qd, 4 w	USA	DR^+^38-PD1-CD4% ↓, CD38^+^CD8^+^ T cells ↓, CD38, HLA-DR, PD-1% on CD4 or CD8(-), monocyte activation (-); CRP (-)	[112]
Probiotics	18-80 years, without history of drug failure	Probiotics (*n* = 20)/healthy donors (*n* = 11)	1 g(a), 48 w	Italy	CD4^+^CD38^+^HLA-DR^+^ T cell ↓, CD8^+^CD38^+^HLA-DR^+^ T cells ↓, IL6 ↓, CRP ↓, sCD14 (-), D-dimer (-)	[109]
Recombinant lactoferrin	≥ 40 years, ART > 1 year, HIV RNA level < 200 copies/mL	A rhlactoferrin then placebo sequence (A-P, *n* = 28), placebo then rhlactoferrin (P-A, *n* = 26)	A-P: M1-3 rhlactoferrin,1500 mg bid, M5-8 placebo P-A: M1-3 placebo, M5-8 rhlactoferrin,1500 mg bid	US	sCD163 ↓, IL-6 (-), D-dimer (-), sCD14 (-), CD8^+^PD1^+^% (-), CD8^+^KI67^+^% (-), D8^+^CD38^+^HLADR^+^% (-), CD4^+^PD1^+^% (-), CD4^+^KI67^+^% (-), CD4^+^CD38^+^HLADR^+^% (-)	[116]
Synbiotics	18-65 years, ART-naïve (stage A or B), CD4 T > 350 cells/*μ*L	Probiotic (*n* = 5), synbiotic (*n* = 5), prebiotic (*n* = 5), placebo (*n* = 5)	Lactobacillus rhamnosus HN001+Bifidobacterium lactis Bi-07 at 10^9^ cfu/mL as probiotics, 10 g of agave inulin as prebiotic, and the combination of both as symbiotic	Mexico	IL-6 ↓, TNF-*α* (-), IL-1*β* (-), IL-10 (-)	[113]
Hypoglycemic agents						
Metformin	Age > 45 years, stable > 1 year on ART, HIV RNA < 50 copies/mL	Metformin (*n* = 6)/control (*n* = 6)	Metformin 500 mg, 24 w	Hawaii	PD1^+^ ↓, PD1^+^TIGIT^+^ ↓, PD1^+^TIGIT^+^TIM3^+^CD4 T ↓	[119]
Sitagliptin	cART for the prior 6 months, CD4^+^ T cell count ≥ 300 cells/*μ*L, HIV RNA < 100 copies/mL	Sitagliptin 100 mg/d (*n* = 18)/placebo (*n* = 20)	100 mg, qd, 8 w	US	hsCRP ↓, CXCL10 ↓, CD4^+^/CD8^+^ ratio (-), D-dimer (-), IL-6 (-)	[122]
Other drugs						
Aspirin	ART for ≥48 w, HIV RNA below quantification limit for ≥48 w	HIV (*n* = 15)/control (*n* = 14)	Aspirin: 81 mg, clopidogrel: 75 mg 24 w	US	sCD14 ↑, sCD163 (-), D-dimer (-), sTNFR1 (-), sTNFR2 (-), sIL-6 (-), thrombogenicity (-), sCD14 (-)	[124]
Celecoxib	18–65 years, asymptomatic, HIV-1-positive patients off ART (HIV RNA > 6000 copies/mL, CD4^+^ T cell count > 300 cells/*μ*L)	Celecoxib arm (*n* = 17)/control arm (*n* = 12)	400 mg, bid, 12 w	Norway	CD38^+^CD8^+^ T% ↓, IgA ↓, a combined score for inflammatory markers ↓, PD-1^+^CD8^+^ T% ↓, CD3^+^ CD4^+^ CD25^+^ CD127^low/−^ Treg ↑	[125]
Cannabis	HIV-1-infected ART-treated participants	Control (*n* = 128) Moderate (*n* = 40) Heavy (*n* = 14)	Moderate (cannabis: 5.1-69.9 *μ*g/L), heavy users (cannabis: ≥70 *μ*g/L)	USA	HLA-DR^+^CD38^+^CD4% ↓, HLA-DR^+^CD38^+^CD8% ↓, CD14^++^CD16^−^% ↑, CD11c^+^CD123^–^ ↓	[129]
Pyridostigmine	No ART, D4 T cell counts ≥ 300/*μ*L	Pyridostigmine (*n* = 9)/placebo (*n* = 10)	30 mg tid, 1 w	Mexico	CD69CD4 ↓, Treg ↑, T cell proliferation ↓, IFN-*γ* ↓, TNF ↓, IL4/5/10 ↑	[131]
Lisinopril	HIV VL < 40-75 copies/mL, CD4^+^T < 350 cells/*μ*L in INRs and ≥350 cells/*μ*L in IRs	Lisinopril (*n* = 16)/placebo (*n* = 15)	Lisinopril 20 mg, 24 w	US	CD38^+^HLA-DR^+^ CD4^+^ (-), CD38^+^HLA-DR^+^ CD8^+^ (-)	

VL: viral load; INRs: immunologic nonresponders; IRs: immunologic responders; LDL: low-density lipoprotein.

## Data Availability

The data used to support the review are included within the article.
